# Two Cardiac Arrests that Occurred after the Administration of Sugammadex: A Case of Kounis Syndrome

**DOI:** 10.1155/2020/6590101

**Published:** 2020-02-17

**Authors:** Machi Yanai, Koichi Ariyoshi

**Affiliations:** Department of Emergency Medicine, Kobe City Hospital Organization, Kobe City Medical Center General Hospital, Kobe, Japan

## Abstract

Kounis syndrome is a form of acute coronary syndrome caused by allergic reactions. No cases of cardiac arrest caused by Kounis syndrome that arose after the administration of sugammadex have been reported. A 71-year-old female suffered two cardiac arrests. The first occurred after sugammadex was administered at the end of an operation for a right radial distal fracture. The patient was resuscitated and transferred to our intensive care unit. She was subsequently discharged home. Five months later, she suffered a second cardiac arrest after sugammadex was administered at the end of an operation for a right femoral neck fracture at our hospital. Urgent coronary angiography revealed multiple coronary spasms. Kounis syndrome was diagnosed based on the patient's elevated serum trypsin levels and a positive result in a skin allergy test of sugammadex. In cases of cardiac arrest with unclear etiologies, Kounis syndrome should be considered.

## 1. Introduction

Kounis syndrome is a rare, but important, cause of acute coronary syndrome (ACS). It is caused by a hypersensitivity reaction after exposure to an allergen [1, 2]. Sugammadex (Bridion®) is a drug that reverses the effects of the neuromuscular-blocking agents rocuronium and vecuronium. Although it is considered to be a safe and well-tolerated drug, concerns about the risk of drug-induced hypersensitivity reactions to sugammadex have emerged from case reports [3–5]. Among case reports about cardiac arrests that occurred after the administration of sugammadex, no cases of cardiac arrest related to Kounis syndrome induced by sugammadex have been reported [6, 7].

We present the case of a patient who suffered two cardiac arrests after the injection of sugammadex. We confirmed that she had suffered coronary vasospasms due to type 1 Kounis syndrome, which was induced by sugammadex.

## 2. Case Presentation

A 71-year-old, 65 kg female with no known history of allergies was transferred to our hospital for postresuscitation care following an in-hospital cardiopulmonary arrest. She had been diagnosed with paroxysmal atrial fibrillation and an old cerebral infarction with no sequelae. Five days before the transfer, she fell and broke her arm. Open reduction and internal fixation were performed at an orthopedic clinic. General anesthesia was induced with 40 mg propofol, 30 mg pentazocine, and 30 mg rocuronium and maintained with 1.5% sevoflurane and nitrogen monoxide (2 L/min). The operation was completed uneventfully within 68 minutes. After the recovery of consciousness, 200 mg of sugammadex was administered, and the patient was extubated. At extubation, her blood pressure was 129/85 mmHg and her pulse rate was 100 beats per minute. Two minutes later, her oxygen saturation (SpO_2_) level suddenly dropped. Mask ventilation was performed. Thirteen minutes later, ventricular fibrillation (VF) occurred, and chest compressions and defibrillation were performed immediately. She was reintubated without difficulty and resuscitated after 18 minutes of resuscitation. She was transferred to our emergency department (ED).

**Figure 1:**
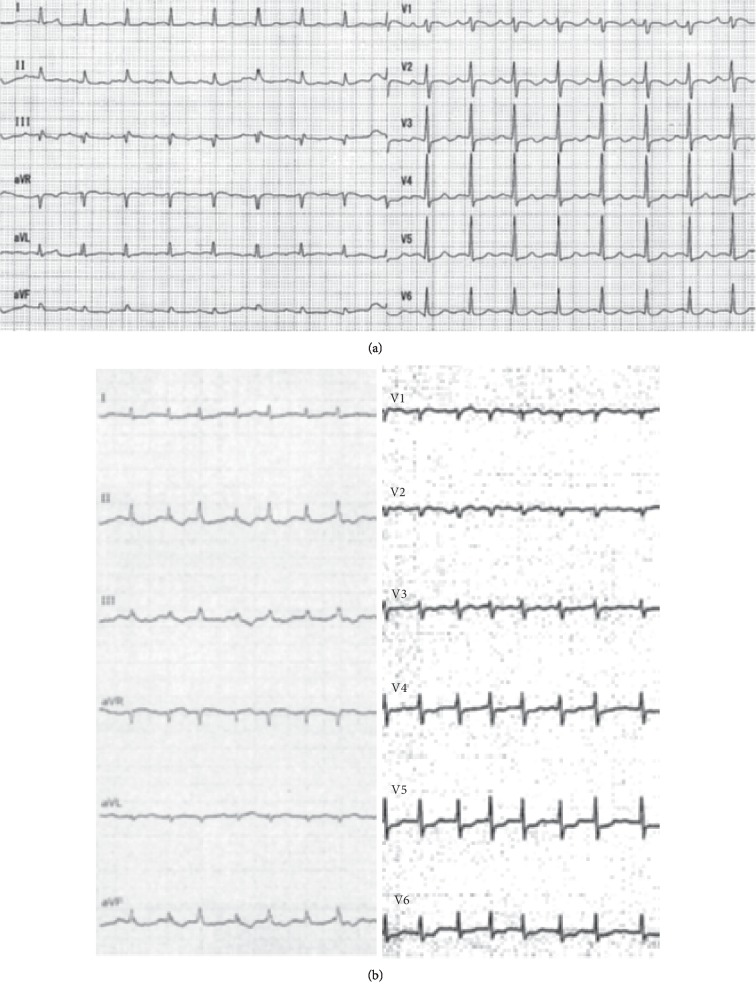
Electrocardiogram (ECG) after 1^st^ and 2^nd^ cardiac arrest. (a) ECG at ER after the 1^st^ cardiac arrest. It showed sinus tachycardia without specific ST segment elevation or depression. (b) ECG at operation room after the 2^nd^ cardiac arrest. It demonstrated diffuse ST depression.

Upon arrival at the ED, she was unconscious (Glasgow Coma Scale: E1VTM1) without sedation. Her blood pressure was 100/60 mmHg, her pulse rate was 100 beats per minute, her respiratory rate was 20 beats per minute with spontaneous breathing, her body temperature was 36.1°C, and her SpO_2_ level was 100% while breathing 100% oxygen. No rash on the skin was observed. A 12-lead electrocardiogram (ECG) showed sinus tachycardia without specific ST segment elevation or depression (Figure 1(a)). Transthoracic echocardiography (TTE) demonstrated a normal ejection fraction with no regional wall motion abnormalities. Blood tests, a chest X-ray, a computed tomography (CT) scan of the brain, and contrast-enhanced CT scans of the chest and abdomen did not produce any remarkable findings. A cardiac troponin I level measured approximately 1 hour after the cardiac arrest was 0.219 ng/mL (reference range (ref): 0–0.028 ng/mL).

In the intensive care unit (ICU), targeted temperature management was initiated. Tracheostomy was performed on day 12 and she was discharged from ICU on day 14. Coronary CT angiography performed on day 45 did not reveal any marked coronary artery disease. Based on the discussion with family members, no further investigations were performed. The patient was transferred to a rehabilitation hospital on day 61.

**Figure 2:**
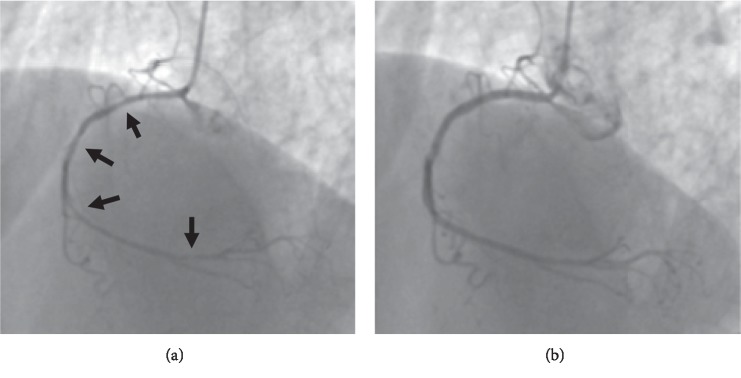
Coronary angiography after 2^nd^ cardiac arrest. (a) Left anterior oblique view demonstrating spastic right coronary artery (arrows). (b) Left anterior oblique view after intracoronary nitroglycerin showing resolution of spasm.

With rehabilitation, she became ambulant and was discharged home. Five months later, she fell and was transported to our ED, being diagnosed with a right femoral neck fracture. Partial hip replacement surgery was planned for the following day. General anesthesia was induced with 0.15 mg fentanyl, 50 mg rocuronium, and 190 mg thiamylal and was maintained with 1% sevoflurane and remifentanil (0.02–0.08 *µ*g/kg/min). The surgery was completed uneventfully within 108 minutes. Two minutes after the administration of 130 mg sugammadex, the patient's blood pressure suddenly dropped. An intravenous noradrenaline drip was initiated, but the patient developed worsening bradycardia and hypotension, before suffering a pulseless electrical activity cardiac arrest. Spontaneous circulation was reinstated after 13 minutes of resuscitation. An ECG demonstrated diffuse ST depression (Figure 1(b)). TTE revealed diffused, severely depressed left ventricular wall motion. An urgent coronary angiogram showed multiple spasms in the right coronary artery, which were resolved via the intracoronary administration of nitroglycerin (Figure 2). The patient's troponin I level was elevated (0.245 ng/mL). As ACS followed by cardiac arrest occurred immediately after the infusion of sugammadex, sugammadex-induced Kounis syndrome was strongly suspected. At 72 minutes after the second cardiac arrest, the patient's tryptase level was elevated (81.2 *µ*g/L; ref: 1.2–5.7 *µ*g/mL). She was admitted to the ICU and treated with drip infusion of 40 mg of methylprednisolone and continuous infusion of nicorandil. She had fully recovered by day 3 and so was extubated. She was discharged from the ICU on day 5. A skin prick test performed on day 13 revealed a positive reaction to sugammadex, i.e., a wheal measuring 5 mm in diameter developed in response to sugammadex, a negative saline control produced negative results, and a positive histamine control caused a 7 mm wheal. Based on the results of the coronary angiogram and the detection of elevated serum tryptase levels and a positive response to a skin allergy test of sugammadex, we concluded that the patient had suffered a cardiac arrest due to type 1 Kounis syndrome induced by sugammadex. She was discharged to a rehabilitation hospital on day 26. She is now living at home and is doing well as an outpatient with mild cognitive impairment.

## 3. Discussion

Cardiovascular allergic reactions have been described for more than 70 years [2]. Kounis syndrome was first described in 1991, which was defined as ACS associated with mast-cell and platelet activation in the setting of allergic insults [1]. Mast cells activate macrophages and T-lymphocytes and release inflammatory mediators, such as histamine, tryptase, and cytokines [1, 2]. These mediators induce coronary vasospasm and sometimes degrade collagen caps, leading to plaque rupture [1, 2]. Type 1 Kounis syndrome involves patients with normal coronary arteries, in whom allergic reactions trigger coronary artery spasms. Type 2 affects patients with pre-existing coronary artery disease (CAD), in whom allergic reactions can cause plaque rupture. Type 3 occurs in patients with pre-existing CAD that have undergone drug-eluting stent placement [1]. Various factors, such as drugs, insect bites, food, and environmental factors, can trigger Kounis syndrome, but drugs are one of the most common triggers [2]. Elevated serum levels of tryptase, one of the proteases released from mast cells, is a useful indicator of Kounis syndrome because the half life of tryptase is longer than those of other mediators. Cardiac arrest occurs in 6.3% of cases [2].

Sugammadex is a selective binding agent, which targets aminosteroid and nondepolarizing neuromuscular-blocking agents. Since its release in Japan in 2010, it has been widely used not only in operation rooms but also in ED and ICU. The Japanese Society of Anesthesiologists (JSA) declared that the incidence of anaphylaxis associated with sugammadex was 0.0029% [8]. However, a recent single-center observational study revealed that the incidence of sugammadex-induced anaphylaxis was 0.039%, which is 13 times higher than the incidence reported by the JSA [8]. Although a systematic review comparing sugammadex with neostigmine concluded that sugammadex caused significantly fewer composite adverse events (relative risk: 0.60), serious allergic reactions, such as Kounis syndrome, were not mentioned [9].

Our patient was diagnosed with Kounis syndrome after her second cardiac arrest. The etiology of the first cardiac arrest was not clear. As the patient initially exhibited VF, and the second cardiac arrest was confirmed to involve sugammadex-induced Kounis syndrome, it is reasonable to suspect that the first cardiac arrest was also caused by an allergic reaction triggered by sugammadex. The cause for preceding desaturation was not clear. It might have been indicative of anaphylactic upper airway obstruction, but she did not recover after the intubation. It indicates that upper airway obstruction was not the main cause of the cardiac arrest. If a sugammadex allergy had been confirmed in the first episode, sugammadex would not have been used again and the second cardiac arrest could have been avoided.

## 4. Conclusion

We reported a case of cardiac arrest caused by Kounis syndrome type 1, which was induced by sugammadex. In cases of cardiac arrest with unclear etiologies, we should always consider anaphylactic reactions, such as Kounis syndrome, and make an effort to find the causative allergens to prevent a second event.
